# Changing the Chevreul Illusion by a Background Luminance Ramp: Lateral Inhibition Fails at Its Traditional Stronghold - A Psychophysical Refutation

**DOI:** 10.1371/journal.pone.0026062

**Published:** 2011-10-13

**Authors:** János Geier, Mariann Hudák

**Affiliations:** 1 Stereo Vision Ltd., Budapest, Hungary; 2 Faculty of Natural Sciences, Department of Cognitive Science, Budapest University of Technology and Economics, Budapest, Hungary; 3 Hungarian Academy of Science-Budapest University of Technology and Economics Research Group of Cognitive Science, Budapest, Hungary; Dalhousie University, Canada

## Abstract

The Chevreul illusion is a well-known 19^th^ century brightness illusion, comprising adjacent homogeneous grey bands of different luminance, which are perceived as inhomogeneous. It is generally explained by lateral inhibition, according to which brighter areas projected to the retina inhibit the sensitivity of neighbouring retinal areas. Lateral inhibition has been considered the foundation-stone of early vision for a century, upon which several computational models of brightness perception are built. One of the last strongholds of lateral inhibition is the Chevreul illusion, which is often illustrated even in current textbooks. Here we prove that lateral inhibition is insufficient to explain the Chevreul illusion. For this aim, we placed the Chevreul staircase in a luminance ramp background, which noticeably changed the illusion. In our psychophysical experiments, all 23 observers reported a strong illusion, when the direction of the ramp was identical to that of the staircase, and all reported homogeneous steps (no illusion) when its direction was the opposite. When the background of the staircase was uniform, 14 saw the illusion, and 9 saw no illusion. To see whether the change of the entire background area or that of the staircase boundary edges were more important, we placed another ramp around the staircase, whose direction was opposite to that of the original, larger ramp. The result is that though the inner ramp is rather narrow (mean = 0.51 deg, SD = 0.48 deg, N = 23), it still dominates perception. Since all conditions of the lateral inhibition account were untouched within the staircase, lateral inhibition fails to model these perceptual changes. Area ratios seem insignificant; the role of boundary edges seems crucial. We suggest that long range interactions between boundary edges and areas enclosed by them, such that diffusion-based models describe, provide a much more plausible account for these brightness phenomena, and local models are insufficient.

## Introduction

**Figure 1:**
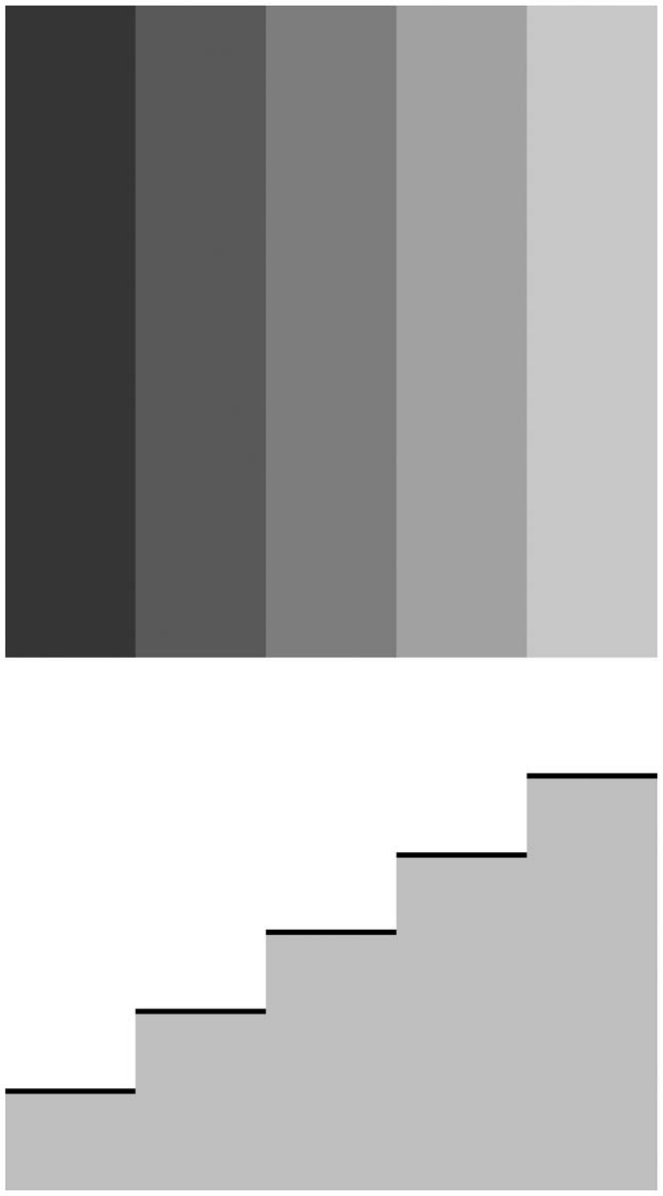
The classical Chevreul illusion. The steps adjacent to each other are physically homogeneous; however, they seem inhomogeneous (crimped). The side of each step adjoining a brighter step seems darker than its other side. The physical luminance cross-section of the midline of the staircase is displayed in the bottom part of the figure.

The Chevreul illusion comprises spatially uniform grey bands of different luminance, which seem inhomogeneous, as if they were crimped: each band looks darker on one side and brighter on the other (see Figure 1). This illusion is attributed to Michel Eugène Chevreul (1786–1889), who, on developing his theory of colour, placed spatially uniform bands of gradually increasing luminance next to each other, whereby he discovered the illusion. Since the physical luminance-cross section profile of this image looks like a staircase, we will use the term ‘staircase’ in this paper for the series of bands, while the bands themselves will be termed as ‘steps’.

Traditionally, the Chevreul illusion has been explained in terms of lateral inhibition, which means that brighter areas projected to the retina inhibit the sensitivity of neighbouring retinal areas. In neurological terms, “cells in one region inhibit cells in adjacent regions” ([1] p2042). In line with this classical principle, the reason for the perceived inhomogeneity in the physically homogeneous steps is that the side of each step neighbouring a lighter one receives more inhibition than its other side.

Lateral inhibition not only serves as the explanatory principle for the Chevreul illusion, but it has long been considered as the basic mechanism of early vision [2]. It stems back as early as the 19^th^ century, since it seemed to explain many of the then known brightness illusions, such as the Hermann grid illusion [3], Mach bands [2], [4], or the simultaneous brightness contrast.

By the 1950s, neuroscientists were searching for lateral inhibition in the visual system of animals, embodied by the circularly symmetric antagonistic (on/off or off/on) retinal receptive fields [5], [6]. Antagonistic circular receptive fields implementing lateral inhibition in the retina are described mathematically by the DoG (Difference of Gaussians) model [7].

By the 1960s, lateral inhibition was considered as a general working principle of sensation in the nervous system [4], and was not limited to visual perception. The principle of lateral inhibition was also adopted by textbooks, and is included in even current ones e.g. [8], [9]. Textbooks demonstrate lateral inhibition as "the working mechanism" of early vision. They illustrate lateral inhibition or the DoG model by means of two classical illusions, the Hermann grid illusion and the Chevreul illusion.

It has to be noted here that many textbooks e.g. [8], [9] misdescribe the Chevreul illusion as Mach bands. The inferential reason for this misdescription is that Mach produced various images by means of quickly rotating disks [2], [4]. Among these figures, there was one that comprised spatially uniform concentric rings of gradually increasing luminance. Although that figure could be regarded as the concentric disk-shaped counterpart of the Chevreul illusion, this, according to Ratliff or von Békésy [2], [4], was not the main image that Mach created. According to these two resources, Mach bands are seen when the linearity of the luminance ramp, which progresses from the centre of a disk towards its edge, breaks. The investigation of Mach bands is not subject of this paper; it has been mentioned only to clarify the terminology misused in some textbooks.

Several current multiscale spatial filtering models of brightness perception also build upon the DoG model with more or less supplementation, retaining its local nature. These theories consider the illusion as a direct consequence of the convolution of the input image with a series of certain DoG-like weight functions e.g. [10]–[12]. All these models vary the DoG principle so that they either use series of DoG filters or their variants, with an elongated shape (ODOG), of various spatial frequencies.

The above-mentioned group of brightness phenomena, which are traditionally explained by lateral inhibition, are also termed contrast phenomena. The basis of this term is that in these images, the perceived contrast is enhanced compared to the physical contrast, as it can be experienced e.g. in the Chevreul illusion at the edges of the steps.

Nonetheless, the Bezold illusion [13], for example, is known already since the 19^th^ century, which cannot be explained by the classical lateral inhibition principle. (The Bezold effect is defined by Gilchrist ([14], p114) as follows “… von Bezold (1874) described and illustrated an effect in which a colored surface appears lighter when overlaid with by thin white lines or small white dots and appears darker if the lines or dots are black.”) The fact that lateral inhibition cannot be considered as the only principal mechanism of early vision is shown more unequivocally by the White effect [15] published in 1979. This illusory effect decisively contradicts the classical lateral inhibition account. In White's figure, grey areas that are surrounded by more white seem brighter than those surrounded by more black, though physically they are of equal luminance. Such phenomena have been termed assimilation in the literature, in order to distinguish them from contrast phenomena. (The term ‘reverse contrast’ is occasionally used as a synonym of the term ‘assimilation’, see for example [16]).

Attempts are found in the literature to capture these two different types of phenomena within a unified computational model framework [11], [12], combining output images of DoG-like local filters. Another attempt for the resolution of this issue is to trace assimilation phenomena back to contrast phenomena by applying certain gestalt grouping principles [17].

In addition to the assimilation phenomena, further images were created to challenge the lateral inhibition account [1], [18]. These novel images were presented to show the role of mid-level mechanisms, involving contours, junctions and grouping in brightness perception [19]. In those studies, novel illusory images were designed in which some parts could be perceived as a dark, semi-transparent smoked glass, shadow or as clouds. The conclusion of these studies was that in the images they presented, identically bright grey areas seemed different because one grey area was interpreted as being located in a shaded area or behind a smoked glass, while the other was perceived as being in a better-lit environment; or as dark disks behind white clouds and vice versa. These authors rejected the lateral inhibition account.

Despite all these counter-examples and arguments, lateral inhibition still persists as a basic explanatory principle. Presumably, theorists of lateral inhibition succeeded in avoiding confrontation with the contradictory phenomena because the mentioned previous studies, that aimed to overthrow the concept that brightness illusions were manifestations of lateral inhibition, applied different illusory images from those that were traditionally explained so. Therefore the idea could still hold true. Most classical illusions known since the 19^th^ century were still in agreement with lateral inhibition-based accounts.

Our general aim is to prove that lateral inhibition (and thus any DoG-based convolution model) is untenable even for the classical illusions. We recently refuted that such models were suitable to explain the Hermann grid illusion (Geier, Sera, Bernath, 2004, *Perception* 33, supplement 53); [20], which, besides the Chevreul illusion, had been considered one of the last strongholds of the lateral inhibition account.

We now show that such local models building upon lateral inhibition fail to explain the Chevreul illusion, too.

## Results and Discussion

A decisive challenge for the lateral inhibition as an explanatory principle for the Chevreul illusion is aimed at by means of the images and phenomena presented below.

### Chevreul staircase surrounded by a luminance ramp background

We placed the Chevreul staircase in a gradually increasing luminance ramp background. (This background is termed as ‘ramp’, since its physical luminance cross-section looks like a ramp.)

Our first main result is that this modification considerably affected the illusion: the illusion significantly increases or decreases, depending on the progression of the ramp relative to the staircase. When the progression of the staircase is identical to that of the ramp, the illusion is enhanced, whereas when the staircase and the ramp progress in opposite directions, the illusion ceases.

**Figure 2:**
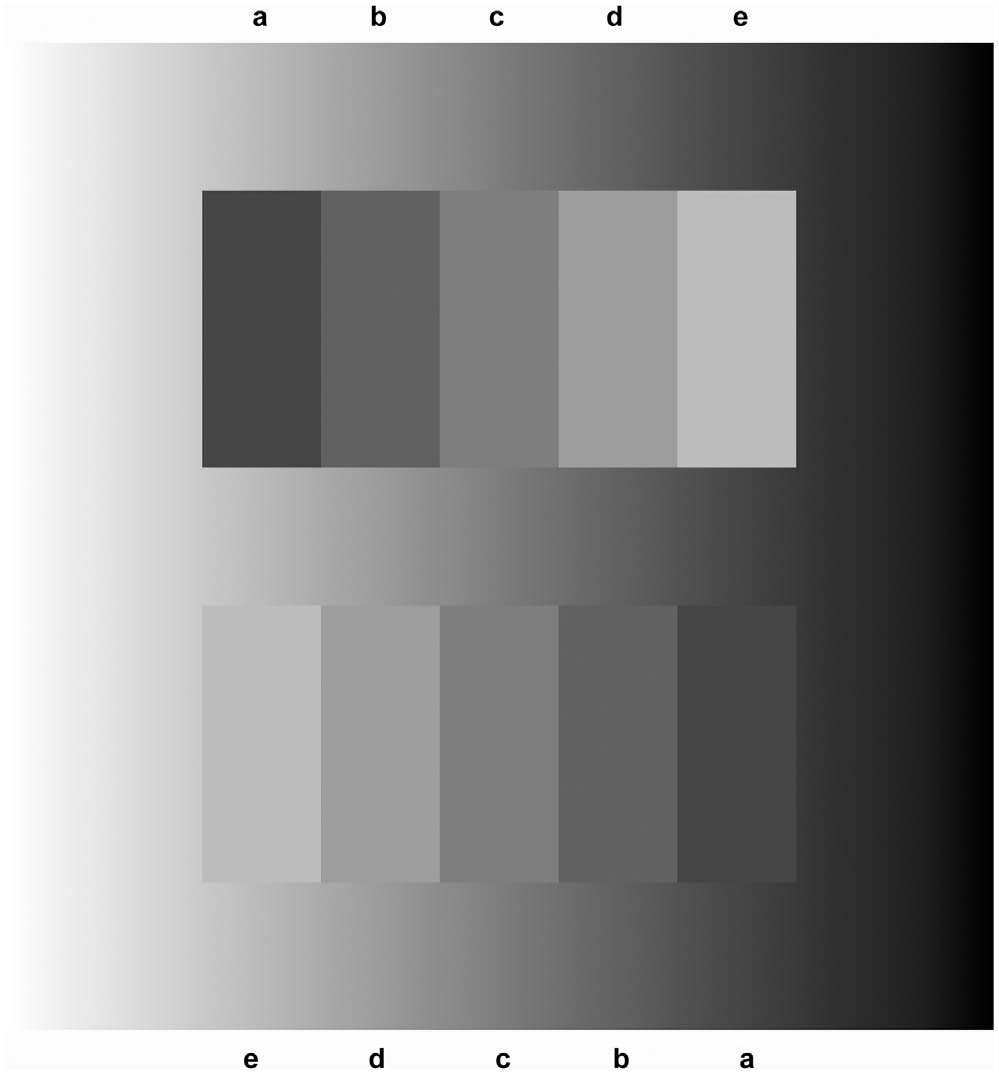
The effect of the luminance ramp background. Two physically identical Chevreul staircases of opposite progression were placed in a luminance ramp background. (Identical letters indicate the steps of physically identical luminance). It can be seen that due to the ramp, the illusion has significantly changed: The illusion ceases if the progression of the staircase is opposite to that of the ramp (upper staircase), while it is strongly enhanced when the progressions of the ramp and that of the staircase are identical (lower staircase).

This phenomenon can be experienced directly by the reader of this paper on looking at Figure 2, where we placed two physically identical staircases of opposite progressions in a luminance ramp background. Note that the change in the illusory effect is equally strong through the entire area of the staircase; it is not limited to the immediate neighbourhood of the upper and lower edges of the steps, where they adjoin the ramp.

The placement of the staircases into a luminance ramp can also be conceived as replacing the originally uniform background (which usually is a white paper) with a luminance ramp background, leaving the staircases themselves physically untouched.

The luminance ramp background was created so that the luminance of the ramp equals the luminance of each step at its vertical midline, whereby the sign of the upper and lower boundary changes along its length. This was adjusted empirically, since the change of illusion was strongest with such parameters. Here we are not aiming to investigate in detail the case when the progression of the ramp is identical to that of the staircase but it is matched to the steps in a different way. We cover this issue only to the extent that we include some such variations in Figure S1).

For the sake of a more exact analysis, we conducted psychophysical experiments with 23 participants. Stimuli used in our experiment are illustrated in Movie S1 and are described in the Materials and Methods section in detail. In the first part of our experiment, we asked the observers whether they saw the steps as crimped (inhomogeneous) or uniform (homogeneous). When the background was homogeneous grey (similarly to the classical demonstration of the Chevreul illusion, as in Figure 1.) 14 observers reported the steps of the staircase as looking crimped, while 9 reported them as uniform. In comparison, when the staircase was surrounded by a ramp of identical progression, all 23 observers reported seeing the steps as crimped. However, when the progression of the ramp was in the opposite direction to that of the staircase, all observers saw the steps as uniform.

Our first conclusion is that if classical lateral inhibition-based explanations were tenable, then the perception within the steps should not have been changed by the ramp. Note that the replacement of the original white background with a luminance ramp background causes physical luminance chance exclusively *outside* the area of the staircase, while no physical change has occurred within the staircase. Classical lateral inhibition-based explanations [2], [8], [9], however, build exclusively upon luminance relations of the steps *within* the staircase. This is in contradiction with the phenomenon that the perception has changed through the entire vertical height of the staircase merely due to the surrounding luminance ramp.

The ramp effect can neither be explained by the mentioned theories of mid-level mechanisms [1], [18], since no physical brightness change occurred within the staircase that could be interpreted as a smoked glass or shadow, nor can any gestalt idea be applied, which could account for the perceptual difference between the two identical staircases of opposite direction in the same ramp background.

### Chevreul staircase surrounded by a double luminance ramp background

If we aim to find a new explanatory principle for these phenomena, we have to notice that due to placing the ramp around the staircases, not only the area outside the staircases has been changed physically, but their boundary edges, too. To decide which of these plays more important role in the change of the Chevreul illusion, we placed another, narrow ramp around the staircase, whose direction was opposite to that of the original, larger ramp.

**Figure 3:**
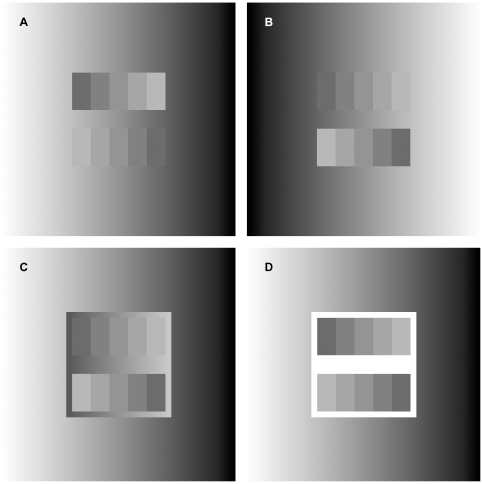
The effect of a double ramp background. The staircase-pairs in the four images are physically identical; the upper and lower staircases in each image are also identical except for their progression to opposite directions. On comparing Fig. 4. A and B, it can be seen that a ramp of opposite direction causes opposite effects. On comparing Figure C with A and B, it can be seen that the illusion in C is identical with that in B. This is so, although the large outer ramp in Fig C is identical with the one in A. Therefore the small inner ramp dominates perception, whose direction is identical to that in B. Finally, the upper and lower staircases in D look identical (except for their direction), therefore here also the inner small area, the homogeneous white rectangle is what dominates.

The result of this modification involving a double luminance ramp can directly be observed in Figure 3. It can be seen there that although the area of the inner ramp is significantly smaller than that of the outer ramp, still the inner one governs the change in the Chevreul illusion. If the inner ramp is replaced by a homogeneous rectangle, then two perceptually identical classical Chevreul staircases will be obtained, progressing in opposite directions, and the outer ramp will have no effect.

For the sake of a quantitative analysis, we supported the effect of the double ramp background by psychophysical experiments. Subjects had to adjust the size of the inner ramp until they found the ramp height at which the steps turned inhomogeneous, if they were uniform at the beginning, or vice versa (see Procedure in Materials and Methods). The changeover occurred at an average height of 0.51 deg above and below the borders of the staircase (SD = 0.48 deg). So, we found that even when the inner ramp is rather narrow, it is still the inner ramp which determines the perceptual experience, whether the steps are seen as strongly inhomogeneous or totally uniform.

This result supports that the upper and lower boundary edges of the staircase control the perceptual experience, and not the area size of the ramp, since such a narrow ramp as half a degree can prevail against the effect of the much larger outer ramp. Therefore, we conclude that it is the boundary edges in the image that govern perceptual experience instead of the large background areas, and long-range interactions should be supposed between edges and the areas enclosed by them.

We summarise the description of these perceptual phenomena as follows:

- *Result 1:* In a Chevreul staircase with a homogeneous background, most observers (roughly two-third of the 23 subjects in our experiment) see the steps as crimped.
- *Result 2:* On placing the staircase in a luminance ramp background of opposite direction, the illusion ceases, while on placing it into a ramp of identical progression, the illusion is significantly enhanced. This was the case for all our 23 observers without exception.
- *Result 3:* When the staircase is placed in a double luminance ramp, the inner one governs the perceptual experience even when its area is rather small compared to the outer one (mean: 0,51 deg), and Result 1–2 also holds here for the perceptual experience.
- *Result 4:* Regardless of the variant of the Chevreul staircase being observed (either the classical one with a homogeneous background or the single or double ramped versions enhancing or ceasing the effect), the extent of the perceived homogeneity (or the inhomogeneity) of each step is equal within the entire height of the staircase. The illusion is of the same magnitude near the upper or the lower boundaries, as well as in the midline of the staircase.

### Lateral inhibition and DoG models

Prior to discussing our criticism in more detail, the concept of lateral inhibition should be further clarified.

On reviewing the relevant literature, two different, but functionally equivalent definitions can be found. One of them has already been used by Ernest Mach: the stimulated neural area inhibits the activity of the neighbouring area. This is termed reciprocal effect by Mach: “…the phenomena discussed can only be explained on the basis of a reciprocal action (Wechselwirkung) of neighbouring areas of the retina” ([2], p97). Mach, for obvious reasons, inferred this on a theoretical basis. The discoverer of lateral inhibition, Haldan Keffer Hartline provided a similar definition ([21], p85), and analogous definitions can also be found in current literature (e.g. [1], p2042).

The other phrasing of the definition emerged presumably after the followers of Hartline (e.g. [6]): a receptive field is associated with each retinal point (or ganglion cell), comprising a stimulating (on) centre and an inhibitory (off) surround. The circularly symmetric on-centre, off-surround DoG (or the Mexican hat) weight function is obtained by the abstraction of physiological measurements [7]. Ratliff ([2] p122) lists the weight functions contrived by six different authors, including the one by Mach himself. Ratliff regards these weight functions fundamentally equivalent. By varying the diameter and the ratio of the stimulating centre and the inhibitory surround, weight functions of different shapes can be produced.

If it is assumed that the decay of lateral inhibition is equal in all directions (isotropy), then the two definitions are practically equivalent. A slight difference between them is that the first phrasing of the definition allows that each retinal point is inhibited by its immediate neighbour, whereas in case of the most widely used DoG filters, this principle is contradicted by the large stimulating centres. Multiscale models attempt to overcome this difficulty by including DoG filters of small diameter.

On this basis, in line with the terminology found in the literature, *we will hereafter identify the concept of lateral inhibition with models using DoG-like filters*, including multiscale models [10], [12], [22], [23] and models using elongated filters [11] as well as any qualitative explanations referring to such, e.g. the classical textbook-explanation.

The aim of the DoG model (as well as other models of brightness perception) is to reproduce the brightness (perceived luminance) distribution from the physical luminance distribution of an image. The input of such a model is an image corresponding to the physical luminance distribution, while another image is expected as output, in which the intensities correspond to human perception.

The main point of DoG-based models is the convolution between the points of the input image and a particular weight function. In other words, the output image is generated by the algorithm from the input image so that each P point of the input image is replaced by the weighted average of the intensities of the neighbouring points of P. The weight function is the given DoG filter, whose central point is allocated at P. In case of multiscale models, a series of DoG (or ODOG) functions are applied, ranging from small to large diameters. Here the output image is the weighted sum of the outputs of individual (O)DoG filters [10]–[12], [22], [23]. Another characteristic of DoG models is that they are local, which means (among other things) that there is no interaction between DoGs (receptive fields) whose centres are located at different points.

### Why is lateral inhibition insufficient here?

The main point of our criticism, as mentioned above, is that the classical lateral inhibition account of the Chevreul illusion considers merely the neighbouring steps as the local surround of each step, and thus it cannot take the effect of the ramp outside the staircase into account. Let us analyze this in more detail.

**Figure 4:**
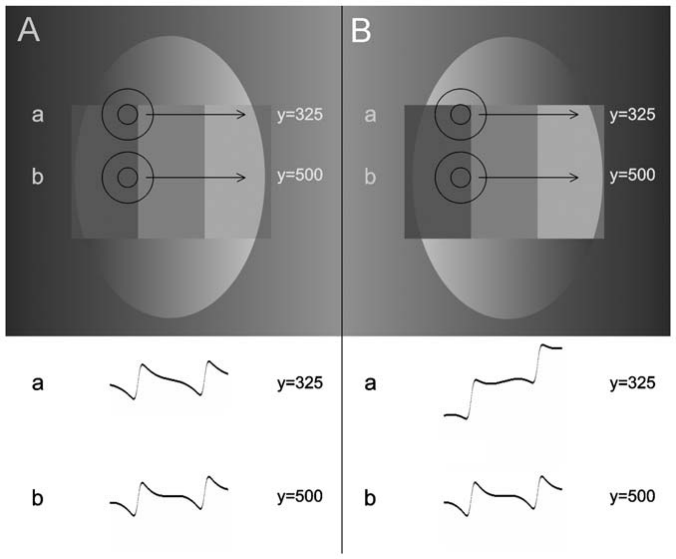
The output of the DoG model for steps in ramps of opposite directions. The middle step of two staircases surrounded by ramps of opposite progressions are enlarged in the upper part of A and B. If the DoG filters are moved along the horizontal direction, as shown by the arrows, they will predict the brightness values shown in the brightness cross-section diagrams *a* and *b* below the image. The luminance cross-sections produced by our simulation of the DoG filter at y = 325 and y = 500 (*a* and *b*) are shown below the enlarged steps, respectively. On the one hand, the prediction of DoG filter (*a*) is somewhat similar to human perception, since it predicts a steeper slope in A. On the other hand, though the step in B is seen as totally flat, DoG filter (*a*) still predicts scalloping there. In addition, in the midline of the two staircases, no difference is predicted between A and B by DoG filter (*b*), contradictory to human perception, according to which the steps in A and B look largely different. Moreover, the predictions of (*a*) and (*b*) within each staircase shows different brightness cross-sections, although the illusion is equally strong through the entire height of the staircase. (The cross-section diagrams were produced by our computer simulation).

DoG filters corresponding to the classical explanation are illustrated in the inner area and near the upper boundary edges of the staircases in Figure 4. By comparing the cross-section diagrams of the responses of DoG filters, two contradictions can be found with human perception. If the cross sections *a* and *b* are compared with each other either within Figure 4A or within Figure 4B, it can be seen that they are significantly different from each other, which contradicts Result 4 (the change of the illusion is equally strong through the entire height of the staircase). Moreover, it can also be seen that the cross-sections *b* of Figure 4A and B are identical, which contradicts Result 2 (ramps of opposite progressions cause opposite effects on the illusion). The cross section diagrams of Figure 4A and B differ only near the horizontal boundary edges of the staircases, showing some similarity to human perception only there: the cross section diagram *a* is steeper in A compared to the one in B. Nonetheless, cross section *a* in B is still crimped, although the steps in B are perceived as uniform.

These contradictions are not surprising, since a significant portion of the inhibitory surrounds of DoGs near the boundary edges (b) reach into the ramp. In contrast, the entire area of DoG filters located in the inner part of the staircase (position (a)) falls only within the staircase, and is not influenced by the ramp.

Another side-effect of such a simple DoG filtering is the blur of the step edges, as it can be seen in the cross-section diagrams. Multiscale models attempt to handle this problem by applying DoG filters of small diameters to avoid blurring, as well as very large ones to ensure that remote points can influence inner parts of large homogeneous areas (e.g. in the ODOG model, the largest filter diameter is 36 deg including the surround). Therefore, it could be reasonable to think that multiscale models can predict the phenomena presented in our images. However, we are going to show below that multiscale filters fail to predict our double-ramped variants for inherent theoretical reasons.

**Figure 5:**
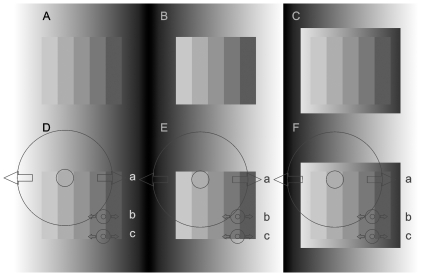
Perceptual experience vs. the stimulation of DoG filters of different spatial scales. The staircases are physically identical in all the six panels. The steps in B are perceived as spatially uniform, while steps in A and C are both perceived as crimped, i.e. the inner ramp dominates in C. Panels D, E and F correspond to A, B and C respectively, illustrating larger and smaller DoG filters at critical locations. A portion of the inhibitory surrounds of small DoG filters near the upper and lower boundary edges of the staircases (*c*) reaches into the ramp, therefore if they are moved along the horizontal direction, their output will be somewhat similar to human perception due to the change of the intensity of the ramp along the horizontal direction. However, if the small DoG filters are moved within the inner area of the staircase (*b*), they do not reach into the ramp, therefore they provide identical outputs for all images, contradictory to human perception. The effect of the ramp background can manifest in the DoG filter outputs in the midline of the staircase if and only if the diameter of the DoG filter is larger than the height of the staircase. Following the same logic as above, DoGs of such large diameters (*a*) might predict the different perception of A and B. Nonetheless, such large filters exceed significantly beyond the inner ramp in F(*a*). As a consequence, the stimulation of DoG filter F(*a*) is much more similar to that of E(*a*) compared to D(*a*). This is in contradiction with human perception, since the perception of A and C are crimped, while B is perceived as flat.

In Figure 5, DoG filters of different diameters are illustrated. In accordance with what was described regarding Figure 4, it can be seen that small DoG filters near the upper and lower boundary edges can produce more or less similar predictions to human perception, since their areas reach into the inner ramp, and do not exceed into the outer one. The small filters in the inner areas of the staircase (Figure 5 D-F DoG *b*), however, produce identical results in A, B and C. Therefore, all in all, the output of small filters contradicts human perception.

If now DoGs of large diameters are considered (Figure 5 D-F DoG *a*), whose inhibitory surrounds extend beyond the staircase into the ramp, it is obvious that these inhibitory surrounds will extend also beyond the narrow inner ramp in Figure 5F into the outer one. Therefore, the stimulation of such large DoG filters in Figure 5F(*a*) will be much more similar to that of E(a) than to that of D(a). Consequently, outputs of large DoGs will reflect a stronger influence of the far surround (outer ramp) than the near surround (inner ramp) in these images.

Nonetheless, the staircases both in A and in C look crimped, whereas the one in B looks flat. Therefore, it is the near surround (inner ramp) that dominates human perception. Consequently, the output of large DoG filters will also be in contradiction with human perception. It also can be questioned whether such large antagonistic, circularly symmetric receptive fields exist.

Since multiscale models use DoGs of diameters ranging from small to large, however, neither small, nor large filters can model the perception of the ramped versions of the Chevreul illusion, the sum of the output images of different scales will also fail to model human perception, irrespective of the averaging method.

The ODOG model [11] must also be mentioned here. In this model, ODOG filters of different orientations are included, whose inhibitory surrounds can roughly be described as elongated ellipses. However, from our point of view, the same criticism stands for elongated ellipses as for circularly symmetric filters: if they are small, then they are insensitive to the ramp in the midline of the staircase while if they are large, then they extend beyond the inner ramp into the outer one, causing it to dominate the simulation results, contradictory to human perception. In conclusion, neither can the ODOG model be expected to predict the perceptual changes in the Chevreul illusion properly.

In the light of the foregoing, it can be stated that DoG models fail to model the novel phenomena. The basic reason of this is that the sensitivity of each DoG filter is limited to the particular area that it covers, however, these critical areas are so various in our images, as it was shown above, that neither small, nor large filters are able to capture these changes, irrespective of whether they are circularly symmetric or elongated.

### Conclusions

On the basis of our results, our conclusions are the following:

- *Conclusion 1:* It is the edges that play the most significant role in the change of the illusion.
- *Conclusion 2:* The edges also obstruct effects coming from farther edges (here the outer edge of the inner ramp prevents the effects coming from the direction of the outer ramp from spreading into the staircase).
- *Conclusion 3:* There is a long range interaction between edges and areas enclosed by them.

These conclusions might extend beyond the Chevreul illusion embedded in background ramp(s). We regard these conclusions generally valid to brightness perception, not being limited to brightness phenomena introduced here.

As it has been shown above in detail, DoG models fail to give a unified explanation to these phenomena. Such models are built on the weighted sum (convolution) of areas covered by single DoG filters, therefore they are essentially sensitive to appropriately weighted average intensities of larger or smaller portions of the image. The accentuated role of edges in the generation of the illusion is not included in DoG models, nor is their segmenting role included. Finally, DoG models do not apply any interaction between filters remotely located from each other.

Let us not be mislead by the fact that the DoG model quasi ‘detects’ edges. This is only a consequence of the DoG model: on the two sides of each edge, areas of two different intensities are found, and the DoG models are in fact sensitive to that. The main point of the concept of lateral inhibition, as it can be found in the definitions of relevant literature, is the reciprocal interaction of neighbouring areas. In these definitions, the role of edges or their effect on larger areas is not even mentioned.

When the principle of lateral inhibition is applied to account for particular illusions, we tend to select areas - that will inhibit each other in accordance with the principle of lateral inhibition – along certain well-discernible edges. Nevertheless, this is a rule that wound its way implicitly to such explanations, which is not contained explicitly by any lateral inhibition model. It is not even forbidden by lateral inhibition models that – ad absurdum – a rectangle is selected mentally without any cue in a uniform white paper, in which case an intriguing contradiction is met: the mentally selected white rectangle should be inhibited by its white surround, implying that the remaining area of the white paper darkens its own inner portion.

By means of the foregoing, we proved directly ‘only’ that the ramped versions of the Chevreul illusion cannot be accounted for by the DoG model. It could be argued against this that the DoG model is still suitable to explain the classical Chevreul illusion presented on a white background. Nonetheless, let us consider the following: by the introduction of the variations with luminance ramp backgrounds, the classical white-backgrounded version has become merely a special case of the broader range of the Chevreul phenomena (i.e. here the slope of the background ramp is 0). It has been shown that the DoG model fails to provide a unified account for the ramped versions, therefore, a new model should be sought. To our knowledge, no such model exists at present in the literature. However, it is certain that if once such a model is developed, it should obviously be able to capture both the ramped versions and the classical Chevreul illusion as well.

If the prediction of a model is more or less agrees with the perceptual facts, it is useful. However, it is not sufficient in itself, since it might happen that this agreement is only apparent, occurring only in a special case. What can be expected from a good model in principle is that it should capture the essence of the modelled process. This is the reason for developing models at all: to understand processes and phenomena better. The DoG model failed to capture the ramped versions, therefore it is clear that it fails to capture the essence of these phenomena. Why would one think therefore that it captures the essence in the classical uniform-backgrounded case?

Therefore, we base our claim that the lateral inhibition-based models are refuted by the ramped versions on the basis of this line of thought. The principle of lateral inhibition is unable to capture the Chevreul illusion since it fails to capture the essence of the broader range of phenomena of which the classical Chevreul illusion is a special case.

Here it is important to note that we do not consider the presence of edges in general a necessary condition for brightness illusions to occur. In other words, if there are edges in an image, then they certainly operate as described in our conclusions. However, this does not exclude the possibility of other brightness illusions, which do not include edges, or if they do, the illusion is influenced by another factor. One example for the latter is Logvinenko's illusion [24], where the effect is caused not by the edges but the sinusoid luminance grating located between the edges. What is certain is that the second derivative of the sinusoid grating is not zero, which is also true for edges. If a model (either an existing one or a future one) captures non-zero second derivatives appropriately, it should also capture edges, as special cases, appropriately.

### Where do we go from here?

Therefore, we are in want of a model that can universally handle the points we claimed in our conclusions. The most suitable candidates for this are the filling-in type of models. The prototype of such models (the ‘standard diffusion model’) is what Cohen and Grossberg [25] applied in one dimension, and after them, Grossberg and Todorović [26] extended it to two dimensions. This ‘CGT’ model was further developed by others, but as it turns out from Gilchrist's review ([14] p106, p206-207), its basic principle is practically unchanged even until nowadays.

The main point of the CGT model in short is that after the allocation of the edges, the areas enclosed by the edges are filled in by a diffusion process. At the same time, edges are also assigned an obstructive role. These principles are fully in line with our conclusions: it is the edges what govern the process; they also have an obstructive role; and the basis of the long range interactions between the edges and the areas enclosed by them is the diffusion process.

However, Gilchrist's comment ([14] p207), that the CGT model is unable to handle the staircase luminance pattern (i.e. the Chevreul illusion) should be taken into account. Here he exposes the following note by Pessoa et al ([27] p2202) on the CGT model: ‘Perhaps an even greater challenge to filling-in models is a luminance staircase distribution. The “steps” of the staircase presumably block diffusion, and it is not evident how a filling-in model can predict that different steps appear with different brightnesses (since “border contrast” is the same everywhere).’

Therefore, the CGT model is in the need of an essential correction: the issue of the brightness of areas separated by edges should be solved. Hopefully, this correction will sooner or later be achieved by someone. (For instance, this candidate could try adding the contrast of the elementary edge segments to the brightness of the points in the neighbouring area in a skilful way…).

## Materials and Methods

### Ethical statement

The experimental procedure was approved by the Budapest University of Technology and Economics Institutional Review Board #1 – Behavioural and Biomedical. Oral informed consent was obtained from all participants after the nature of the experiment was explained both in written format on the application form and orally before the experiment. The reason for not collecting written consent from each subject is that our experiment had no risk at all and caused no harm. People only had to look at an image displayed on a computer screen and were free to rest or leave anytime, and they were informed so beforehand. The process was documented by our experimental software: name of the subject, age, gender, and type of vision (normal, or wearing glasses or contact lenses). The documented measure was a parameter of the viewed image that the subject set for herself (the height of the inner ramp at which the illusion turned over for her). Only the two researchers have access to the data, which was processed anonymously. The institutional ethics committee approved this process.

### Subjects

23 observers (12 males, 11 females), aged 18-32 years, with normal (20) or corrected-to-normal (3) vision, participated.

### Stimuli

A staircase luminance profile of 3.68*7.38 deg, consisting of 6 steps, was used for a stimulus. Steps were 1.23 deg wide and had luminances of 18.3, 13.9, 10.0, 7.4, 4.5, and 2.5 cd/m^2^. In the first part of the experiment, the staircase was surrounded by (i) a uniformly grey background of 7.3 cd/m^2^; (ii) a smooth luminance ramp ranging from 30.3 – 0.1 cd/m^2^ and progressing either in the same or (iii) in the opposite direction as the staircase. All backgrounds subtended 12.27*12.27 deg. In the second part, a background, consisting of an outer ramp and an inner ramp of opposite progression, surrounded the staircase. The inner ramp was 9.81 deg wide (luminances as stated above (ii)). Four stimuli were used: the staircase progressed either (i) from high to low or (ii) low to high, thus its progression was either the same or the opposite to the inner ramp. Initially, the inner ramp either (iii) surrounded the Chevreul staircase extending by 2.5 deg above and below or (iv) was occluded by the staircase (0 deg visible above and below). Stimuli were presented on a calibrated CRT monitor (resolution 1024*768 pixels, 60 Hz) in a dimly lit room at a distance of 72 cm.

### Procedure

In the first part of the experiment, we tested the effect of the various backgrounds on the Chevreul illusion. Observers were asked whether the individual steps of the staircase appeared either darker on one side and lighter on the other (crimped), or uniform. In the second part, they adjusted the initial size of the inner ramp, until the percept of the steps in the staircase changed from crimped to uniform, or vice versa. The aim was to measure the minimal size of the inner ramp at which it still prevailed over the effect of the outer ramp, in order to determine whether area size proportions or boundary edges were more important. After familiarization with the task, stimuli were presented in a random order, followed by a mask of black-and-white dots exposed for 2500 ms.

## Supporting Information

Figure S1 **The demonstration of the case when the sign of the elementary edge segments changes along the upper and lower boundary edges of the staircase (A3) and the cases when it does not (A1, A2, A4, A5).** The luminance cross-sections of the horizontal midline of the staircases are displayed below each ramped Chevreul image. The three staircases are physically identical within each row. The intensities of the staircases increase from row 1 to 6, whereas the background ramps are identical in all rows. Rows: A1: the intensity of the steps is lower than that of the background ramp. A2: the left side of the steps is fitted to the ramp. A3: the vertical midline of the steps is fitted to the ramp. A4: the right side of the steps is fitted to the ramp. A5: the intensity of the steps is higher than that of the ramp. Columns: A: the progression of the ramp is identical to that of the staircase. B: the progression of the ramp is opposite to that of the staircase. C: white background. It can be seen that it is column A in which the steps are crimped to the highest extent; the illusion is weakest in column B, while the magnitude of the illusion in C is in between that in A and B. It can be seen that the crimping effect of the ramp is strongest in A3, but it is also not negligible even in the other rows. This fact deserves attention particularly because the intensity of the staircases run below the intensity of the ramp in A1, and above it in A5, while the ramp still changes the crimping of the steps. This implies that the change of the sign along the upper and lower boundary edges of the staircase is not a necessary condition for the ramp effect to occur.
(TIF)

Movie S1 **Illustration of our experimental stimuli.**
(SWF)
