# Supplementary figures and images for: Changing the Chevreul Illusion by a Background Luminance Ramp: Lateral Inhibition Fails at Its Traditional Stronghold - A Psychophysical Refutation
Source: PLoS One. 2011 Oct 13;6(10):e26062. doi: 10.1371/journal.pone.0026062 (PMC3192777; doi:10.1371/journal.pone.0026062)

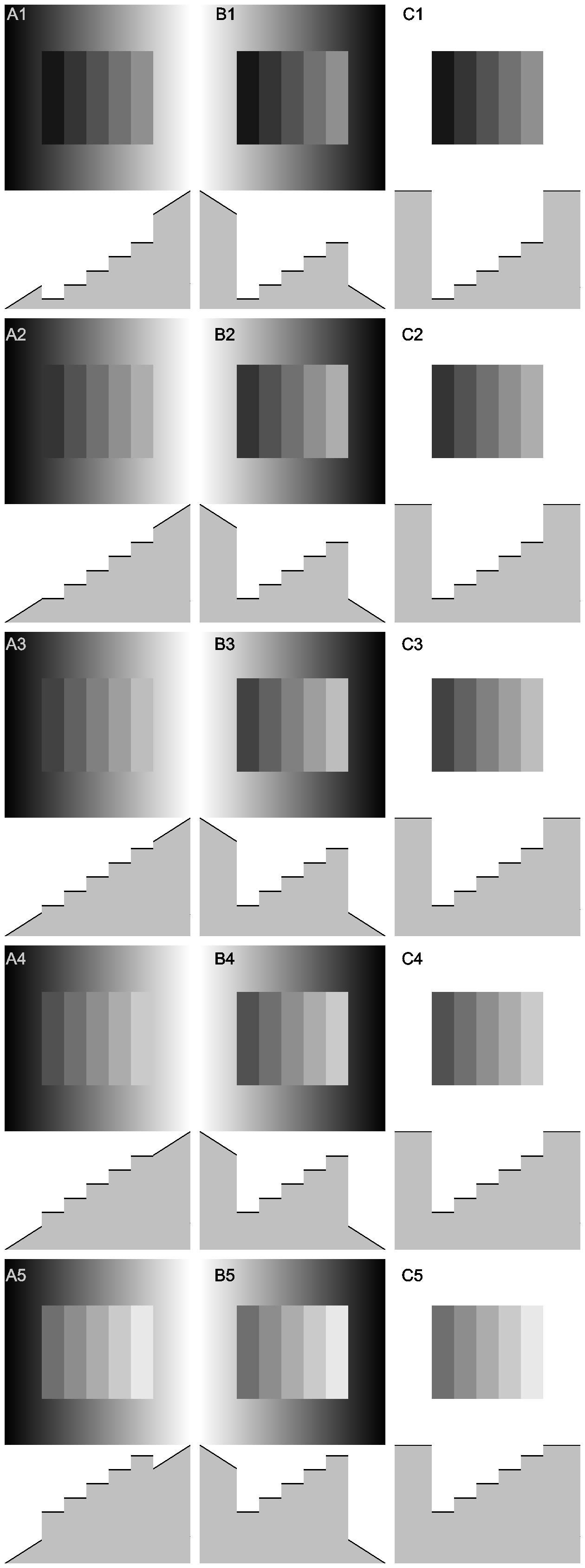

Supplement: Figure S1 — The demonstration of the case when the sign of the elementary edge segments changes along the upper and lower boundary edges of the staircase (A3) and the cases when it does not (A1, A2, A4, A5). The luminance cross-sections of the horizontal midline of the staircases are displayed below each ramped Chevreul image. The three staircases are physically identical within each row. The intensities of the staircases increase from row 1 to 6, whereas the background ramps are identical in all rows. Rows: A1: the intensity of the steps is lower than that of the background ramp. A2: the left side of the steps is fitted to the ramp. A3: the vertical midline of the steps is fitted to the ramp. A4: the right side of the steps is fitted to the ramp. A5: the intensity of the steps is higher than that of the ramp. Columns: A: the progression of the ramp is identical to that of the staircase. B: the progression of the ramp is opposite to that of the staircase. C: white background. It can be seen that it is column A in which the steps are crimped to the highest extent; the illusion is weakest in column B, while the magnitude of the illusion in C is in between that in A and B. It can be seen that the crimping effect of the ramp is strongest in A3, but it is also not negligible even in the other rows. This fact deserves attention particularly because the intensity of the staircases run below the intensity of the ramp in A1, and above it in A5, while the ramp still changes the crimping of the steps. This implies that the change of the sign along the upper and lower boundary edges of the staircase is not a necessary condition for the ramp effect to occur. (TIF) [file pone.0026062.s001.tif]
